# Health-related quality of life among individuals with long-standing spinal cord injury: a comparative study of veterans and non-veterans

**DOI:** 10.1186/1471-2458-10-6

**Published:** 2010-01-05

**Authors:** Soheil Saadat, Masoud Javadi, Baharak Sabet Divshali, Amir Hussein Tavakoli, Seyed Mohammad Ghodsi, Ali Montazeri, Vafa Rahimi-Movaghar

**Affiliations:** 1 Sina Trauma and Surgery Research Center, Tehran University of Medical Sciences, Tehran, Iran; 2 Janbazan Medical and Engineering Research Center (JMERC), Tehran, Iran; 3 Brain and Spinal Cord Injury Repair Research Center (BASIR), Tehran University of Medical Sciences, Tehran, Iran; 4 Department of Mental Health, Iranian Institute for Health Sciences Research, ACECR, Tehran, Iran; 5 Research Centre for Neural Repair, Tehran University, Tehran, Iran

## Abstract

**Background:**

Spinal cord-injured (SCI) patients experience poor health-related quality of life (HRQOL) and they usually report lower HRQOL than the general population or population subgroups in Iran and elsewhere. The aim of this study was to compare HRQOL between veterans and non-veterans with SCI in Iran.

**Methods:**

This was a cross-sectional study. HRQOL was measured using the 36-item Short Form Health Survey (SF-36). Thirty-nine male veterans and 63 non-veteran males with SCI were included in the study. Regression analyses were applied to determine the variables affecting physical and mental health-related quality of life among the patients.

**Results:**

The male veterans had a lower HRQOL than the non-veterans with SCI. The differences were significant for all measures except for physical and social functioning. The greatest difference was observed for bodily pain (P = 0.001). The regression analysis results indicated that a longer time since injury was associated (P = 0.01) with better physical health-related quality of life (PCS), while being a veteran (P < 0.001) and having a spinal lesion in the cervical region (P = 0.001) were associated with poorer PCS. Older age (P < 0.001) and higher education (P = 0.01) were associated with better mental health-related quality of life (MCS), while being a veteran and having a spinal lesion in the cervical region (P = 0.02) were associated with poorer MCS.

**Conclusion:**

The study findings showed that veterans with SCI experienced lower HRQOL than their non-veteran counterparts. A qualitative study is recommended to evaluate why HRQOL was lower in veterans than in non-veterans with SCI although veterans had higher incomes as a result of their pensions and increased access to equipment, and medications. To improve quality of life in both veterans and non-veterans with spinal cord injuries, policy changes or implementation of new interventions may be essential so that veterans could receive additional support (e.g. counseling, recreation therapy, vocational therapy, etc.) and non-veterans could meet their basic needs.

## Background

Spinal cord injury (SCI) causes several health problems that negatively affect not only the patient's physical condition but all aspects of their lives including their goals and communications, and more importantly their health-related quality of life [1-6]. Active young males are the individuals who most commonly experience SCI, and since this disability lasts for the rest of their lives [7], studying quality of life among this population is important.

Overall, the findings from several studies in the USA [8,9], Canada [10], Sweden [11] and Norway [12] using the 36-item Medical Outcomes Short Form Health Survey (SF-36) showed that HRQOL was low among SCI patients. These patients scored lower on almost all the SF-36 subscales or its component summary scores than either the general population or other population subgroups. As expected these patients scored lower on physical functioning as compared to other measures such as social functioning or mental health. The score for this particular domain was 26.6, 19.3, 23.9, 33.0, and 36.3 (out of 100) respectively corresponding to a difference of 25 to 60 points lower than the general population or normative data adjusted for age and gender [8-12]. On occasion, patients with SCI reported a better condition than the general population. For instance, a USA study showed that the mental component summary score of the SF-36 for SCI patients was higher than the normative value for the general population [8]. However, not only is quality of life low among veterans in general, but studies have also shown that they experience poorer HRQOL and overall health status than their non-veteran counterparts [13].

During the 1980-1988 Iran-Iraq war, the human cost to Iran included more than 200,000 lives lost and more than 400,000 persons injured [14], of whom 2012 were veterans with spinal cord injury. Currently, these veterans have access to several services including free healthcare such as bed sore management, wheelchair and rehabilitation services, insurance and special centers for annual physical examinations and laboratory tests, plus 800 US dollars per month to pay for a nurse and about 1200 US dollars monthly salary for their living expenses. In contrast, non-veterans do not have free insurance and only receive incomplete services from the State Welfare Organization of Iran, including a monthly salary of 50 US dollars, a wheelchair and limited rehabilitation services.

This study has two aims: to assess quality of life in male spinal cord injured veterans; and to compare health-related quality of life between veterans and non-veterans with SCI. To the best of our knowledge this is the first paper from Iran that reports on the topic.

## Methods

### Design and data collection

This was a cross-sectional study of quality of life among a convenient sample of SCI veterans and non-veteran SCI patients in Tehran, Iran. Veterans were approached during a recreational trip and were asked if they wished to take part in the study. Thirty-nine out of 41 veterans agreed to be interviewed. Additional information was extracted from their case records kept in the Janbazan (Veterans) Medical and Engineering Research Center. Non-veterans were selected from a consecutive sample of patients attending a neurosurgery outpatient department in Imam Hospital, affiliated to Tehran University of Medical Sciences, during the study period. Eighty-three patients were approached and 63 agreed to take part. A face-to-face interview was arranged to collect data from both groups. Trained staff from the Janbazan (Veterans) Medical and Engineering Research Centre interviewed the veterans and trained staff from the Brain and Spinal Cord Injury Repair Research Center (BASIR) interviewed the non-veterans.

### Measures

Health-related quality of life was measured using the SF-36 questionnaire. The psychometric properties of the Iranian version of SF-36 are well documented [15]. The questionnaire comprises measures of physical functioning, role limitation due to physical problems (RP), bodily pain (BP), general health perceptions (GH), vitality (VT), social functioning (SF), role limitation due to emotional problems (RE) and mental health (MH). These scales also provide two component summary scores: physical component summary (PCS) and mental component summary (MCS). Scores range from 0 to 100 with higher scores indicating better conditions [16,17]. This method of scoring (summated ratings) assumes that item or items belonging to each scale can be transformed or summed without standardization of scores or item weighting. We used this method to calculate scores.

For each individual, we also collected data on demographic factors, history since injury, level of injury, motor lesion, marital status at the time of injury and at the time of the study, and the number of children. It was thought that these variables might have significant positive or negative effects on health-related quality of life. For example, since patients might have suffered from infertility since their injury, this could affect their quality of life; hence we were interested in collecting data on the number of children.

### Statistical analysis

In univariate analysis, the chi-square test (or Fisher's exact test and Yate's correction where necessary) was used to compare categorical variables. To compare the SF-36 scores between veterans and non-veterans, Student's t-test was performed. We estimated regression models to examine the association between individual characteristics and HRQOL. Separate linear regression analyses were carried out for the PCS and MCS, which were treated as dependent variables. For this purpose, the distributions of the PCS and the MCS scores were examined. There was slight negative skewness but this was less than one in both cases, allowing us to using the test. Age, time since injury, being a veteran, educational level, having a spouse, and SCI in the cervical region were included in the model as independent variables. Variables that failed to represent significant P values (P < 0.05) were excluded from the model using a backward elimination procedure.

### Ethics

The study received approval from the Sina Trauma and Surgery Research Center, affiliated to Tehran University of Medical Sciences, and the Janbazan (Veterans) Medical and Engineering Research Center ethics committee. All patients gave their informed consent.

## Results

Thirty-nine veteran and 63 non-veteran spinal cord-injured patients were included in the study. All the patients were male. Table 1 presents the characteristics of the study groups. The etiology of SCI in the veterans was shrapnel wounds (34.3%), bullets (22.9%), mines (5.7%), falls (8.6%), road traffic accidents (2.9%) and other causes (20.0%). In the non-veteran group, SCI resulted from road traffic crashes (49.2%), falls (31.7%), violence (12.7%), sports injuries (3.2%) and other causes (3.2%).

**Table 1:** The characteristics of the study groups

		Veterans (n = 39)	Non-veterans (n = 63)	
		**No. (%)**	**No. (%)**	**P**

**Age (year)**				

	Mean (SD)	45.2 (5.3)	34.3 (8.3)	< 0.001

	Range	35-59	19-57	

**Educational level**				< 0.001

	Secondary school or less	4 (11.1)	36 (57.1)	

	Diploma or college	14 (38.9)	20 (31.7)	

	Higher	18 (50.0)	7 (11.1)	

**Employment status**				0.151

	Employed	12 (34.3)	12 (19.0)	

	Unemployed	23 (65.7)	46 (73.0)	

	Retired	0 (0.0)	5 (8.0)	

**Had spouse at the time of injury**				< 0.001

	Yes	8 (21.6)	38 (60.3)	

	No	29 (78.4)	25 (39.7)	

**Have spouse at the time of study**				< 0.001

	Yes	30 (88.2)	34 (54.0)	

	No	4 (11.8)	29 (46.0)	

**Number of children**				0.006

	0	3 (13.0)	31 (49.2)	

	1	13 (56.5)	11 (17.5)	

	2	5 (21.7)	15 (23.8)	

	≥ 3	2 (8.7)	6 (9.6)	

**Time since injury (year)**				

	Mean (SD)	23.4 (3.6)	7.0 (4.9)	< 0.001

	Range	12-29	1-28	

				

**Level of injury**				0.818

	Cervical	22 (56.4)	37 (58.7)	

	Thoracic/Lumbar	17 (43.6)	26 (41.3)	

**Motor lesion**				< 0.001

	Complete	39 (100)	29 (46.0)	

	Incomplete	0 (0.0)	34 (54.0)	

The SF-36 scores for veterans and non-veterans are shown in Table 2. There were significant differences between the two groups for all measures except for physical and social functioning, indicating that the veterans were experiencing poorer quality of life. All the veterans had complete SCI but the non-veterans had both complete and incomplete SCI. However, when the analysis was restricted to veterans and non-veterans who had suffered complete motor lesions, the results remained unchanged (Table 3).

**Table 2:** The SF-36 scores in veteran versus non-veteran SCI patients

	Veterans (n = 39)	Non-veterans (n = 63)	
	**Mean (SD)**	**Mean (SD)**	**P**

Physical functioning	25.9 (30.3)	34.9 (23.8)	0.115

Role physical	36.2 (37.5)	59.0 (34.8)	0.003

Bodily pain	54.2 (32.3)	75.4 (20.5)	0.001

General health	52.8 (20.9)	62.9 (17.0)	0.009

Social functioning	67.8 (23.9)	75.0 (24.1)	0.145

Role emotional	49.1 (41.8)	74.1 (33.5)	0.003

Vitality	62.4 (15.5)	73.3 (16.3)	0.001

Mental health	67.8 (16.8)	76.0 (15.5)	0.014

Physical component summary (PCS)	42.4 (19.5)	58.1 (14.8)	< 0.0001

Mental component summary (MCS)	62.3 (17.7)	74.6 (16.0)	< 0.0001

**Table 3:** The SF-36 scores in veteran versus non-veteran suffering from complete motor lesions SCI

	Veterans (n = 39)	Complete motor lesions Non-veterans (n = 29)	
	**Mean (SD)**	**Mean (SD)**	**P**

Physical functioning	25.9 (30.3)	27.6 (20.4)	0.78

Role physical	36.2 (37.5)	60.1 (33.7)	0.009

Bodily pain	54.2 (32.3)	77.4 (20.5)	0.001

General health	52.8 (20.9)	67.9 (15.7)	0.002

Social functioning	67.8 (23.9)	75.0 (26.3)	0.25

Role emotional	49.1 (41.8)	74.7 (32.9)	0.007

Vitality	62.4 (15.5)	73.4 (14.7)	0.004

Mental health	67.8 (16.8)	77.0 (14.5)	0.02

Physical component summary (PCS)	42.4 (19.5)	58.2 (13.3)	<0.0001

Mental component summary (MCS)	62.3 (17.7)	75.0 (16.5)	0.004

Finally, regression analyses were applied to determine the effects of variables associated with PCS and MCS. Longer time since injury (P = 0.01) was associated with a better PCS, while being a veteran (P < 0.001) and having a spinal lesion in the cervical region (P = 0.001) were associated with a poorer PCS (Table 4). Older age (P < 0.001) and higher education (P = 0.01) were associated with a better MCS, while being a veteran and having a spinal lesion in the cervical region (P = 0.02) were associated with a poorer MCS (Table 5).

**Table 4:** Regression analysis for physical component summary (PCS)*

Variables	Unstandardized Coefficients		Standardized Coefficients		
	**Beta**	**Std. Error**	**Beta**	**t**	**P**

Non-veterans (versus veterans)	26.764	3.602	0.392	7.431	<0.001

Time since injury	7.695	2.910	0.206	2.645	0.01

Lack of spinal injury in cervical region	10.515	3.074	0.283	3.421	0.001

* Variables that failed to represent significant P values (P < 0.05) were excluded from the model using a backward elimination procedure.

**Table 5:** Regression analysis for mental component summary (MCS)*

Variables	Unstandardized Coefficients		Standardized Coefficients		
	**Beta**	**Std. Error**	**Beta**	**t**	**P**

Non-veterans (versus veterans)	26.703	3.258	0.299	8.197	<0.001

Age	0.857	.144	0.461	5.967	<0.001

Educational level	5.310	2.111	0.145	2.515	0.01

Lack of spinal injury in cervical region	7.277	3.165	0.149	2.299	0.02

* Variables that failed to represent significant P values (P < 0.05) were excluded from the model using a backward elimination procedure.

## Discussion

The study findings showed that male SCI veterans had lower HRQOL than male non-veteran SCI patients in Iran. Both the PCS and MCS and the SF-36 scores (except for physical and social functioning) were lower in veterans. Even comparison of the scores between veterans and the subset of non-veterans (those with complete motor lesions) yielded similar results. It is argued that complete motor lesions may lead to the occurrence of pressure ulcers and other complications by limiting the patient to bed or a wheelchair [18], so they might be associated with poorer HRQOL than patients with incomplete SCI [5,19]. It is argued that since many individuals with incomplete injuries also use wheelchairs and are at high risk for pressure ulcers thus the completeness of injury as a single reason could not justify these differences and there is need to find out more specific reasons and then pay attention to these reasons in order to improve quality of life in veterans. For example a recent study on pressure ulcers in veterans with spinal cord injuries found that diabetes and depressive symptoms were the most significant factors predicting pressure ulcers [20]. However, as indicated in Table 3, physical functioning was very low for both veterans (25.9) and non-veterans (27.6). This might be due to serious motor deficits in both groups. In contrast, the mean scores for role limitation due to physical problems (RP) were very different (36.2 in veterans vs. 60.1 in non-veterans). This means that veterans were more affected by their physical disabilities. Possible explanations for this might be related to differences in age or chronic conditions between the veterans and non-veterans. These possibilities should be investigated further in future studies.

Comparing role emotional (RE) in veterans and non-veterans revealed a significant difference between the two groups. It seems that male veterans with SCI had the more severe emotional disturbances in Iran. In contrast, two other published studies, the first from Iran concerning female veterans with SCI [21], and the second from the USA concerning veterans with SCI [22], showed that veterans scored higher on RE than on other subscales. A comparison of RE between Iranian and American veterans is not sound, and psychotherapy and other facilities might be much better for veterans in the USA, but the very low RE in our study subjects still needs more and deeper evaluation.

It has been shown that frequent mental distress and depressive symptoms are associated with poor self-reported health in individuals with spinal cord injury [23]. In addition, perceived stress was found to be higher in SCI veterans than non-veteran men with SCI. Another study found that male veterans with SCI had more hassles related to financial support, physical ability, health and healthcare in their daily lives; and they were more likely to be homeless, smokers, and heavy drinkers. The important issues acknowledged most frequently by veterans with SCI included physical abilities (endorsed by 70%), their own health (62%), medical care (56%), money for extras (59%), money for emergencies (57%), and money for necessities (56%) [24]. Thus, healthcare personnel providing services to veterans with SCI need to be aware of the hassles and chronic strains affecting their everyday lives. Indeed, comparison of stress and post-traumatic stress disorder (PTSD) between veterans and non-veterans might help to reveal additional reasons for the observed differences in health-related quality of life between these two groups. Unfortunately, we did not collect data on such variables. This should be considered for similar studies in the future.

Higher expectation of life quality among veterans may be another explanation for the lower HRQOL among male veteran SCI patients. In fact, although SCI veterans enjoyed financial support and increased health care access, their higher expectations might have resulted in lower HRQOL than among the non-veteran SCI patients. To explain further, it should be noted that many Iranian veterans with SCI took part in the Iran-Iraq war voluntarily and intentionally, and expected that at the end of the war they would have an ideal society. They were ready to be injured or even to die to establish an ideal country. They self-sacrificed their health, but when they came back to their home towns after their injuries or 8 years of war, they were confronted with a society worse than they expected; a society with many broken values. Thus, their evaluation of their new life was now very different from that of a patient who, for instance, had suffered SCI following a road accident and consequently reported a lower quality of life. We believe this explanation is highly consistent with the quality of life definition offered by the World Health Organization: 'an individual's perception of his/her position in life in the context of the culture and value system in which he/she lives, and in relation to his/her goals, expectations, standards, and concerns' [25]. There is evidence that for example expectations regarding aging could influence physical and mental health. A recent study reported that a positive expectation about ageing was associated with better physical and mental health, after adjusting for age, gender and education [26]. However, this hypothesis is the authors' own thoughts and merits further investigation in future studies of HRQOL among injured veterans in Iran and perhaps elsewhere.

We used a well-known instrument (the SF-36) to measure quality of life in individuals with SCI. This is a generic measure, and it would seem much better to assess quality of life in this population by more specific questionnaires in addition to such instruments. For example, Luther et al. developed an 8-item SCI-specific physical functioning scale for veterans that showed strong psychometric properties [27]. It is argued that little research has been conducted specifically to ascertain perception of quality of life among people with spinal cord injuries [28]. However, as noted in a recent review of the literature, much remains to be discovered about SCI individuals in terms of their functional health, well-being, and the relative burden of the disease [29]. The effect of physical disability or illness cannot be fully understood without considering both the specific areas of functioning affected by the physical condition and the aspects of health-related quality of life that are of particular importance to the individual [30].

This study had several limitations. The major limitations were the small sample size and the fact that we did not collect data on the patients' chronic illnesses and common complications. There is evidence that chronic illnesses have a substantial effect on health-related quality of life for persons with SCI [23,31,32]. In addition, the veterans were much older than the non-veterans so they were probably experiencing higher rates of diabetes, renal disease, chronic pulmonary diseases and other chronic illnesses, or pressure ulcers, than the non-veteran group. Time since injury also was longer for the veterans. Thus, the findings from this study might not be generalized, although two recent studies have shown no relationships between time since injury, age and quality of life among individuals with spinal cord injuries [2,3].

## Conclusion

The study findings showed that veterans with SCI experienced lower HRQOL than their non-veteran counterparts. A qualitative study is recommended to evaluate why HRQOL was lower in veterans than in non-veterans with SCI although veterans had higher incomes as a result of their pensions and increased access to equipment, and medications. To improve quality of life in both veterans and non-veterans with spinal cord injuries, policy changes or implementation of new interventions may be essential so that veterans could receive additional support (e.g. counseling, recreation therapy, vocational therapy, etc.) and non-veterans could meet their basic needs.

## Abbreviations

SCI: Spinal cord injury; HRQOL: Health related quality of life; PCS: Physical component summary; MCS: Mental component summary; SF-36: 36-item Short Form Health Survey questionnaire; RP: role limitation due to physical problems; RE: role limitation due to emotional problems

## Competing interests

The authors declare that they have no competing interests.

## Authors' contributions

SS contributed to the data analysis and writing the first draft. BSD wrote the proposal. AHT, MJ, and SMG confirmed proposal and collected the SF-36 data and took part in the study. AM helped as a consultant in all parts of the study, rechecked the data analysis and revised the final manuscript. VRM was the principal investigator and performed quality control on patients' case records, was responsible in all parts of the study, wrote draft and revised the manuscript. All authors read and approved the final manuscript.

## Pre-publication history

The pre-publication history for this paper can be accessed here:

http://www.biomedcentral.com/1471-2458/10/6/prepub
